# Pre-Surgery Cognitive Performance and Voxel-Based Lesion-Symptom Mapping in Patients with Left High-Grade Glioma

**DOI:** 10.3390/cancers13061467

**Published:** 2021-03-23

**Authors:** Ilaria Guarracino, Tamara Ius, Cinzia Baiano, Serena D’Agostini, Miran Skrap, Barbara Tomasino

**Affiliations:** 1 Scientific Institute, IRCCS E. Medea, Dipartimento/Unità Operativa Pasian di Prato, 33037 Udine, Italy; ilaria.guarracino@lanostrafamiglia.it; 2 SOC Neurochirurgia, Azienda Sanitaria Universitaria Friuli Centrale ASU FC, 33100 Udine, Italy; Tamara.ius@asufc.sanita.it (T.I.); skrap@asufc.sanita.fvg.it (M.S.); 3 Division of Neurosurgery, Università degli Studi di Napoli “Federico II”, 80133 Naples, Italy; cinzia.baiano@unina.it; 4 SOC Neuroradiologia, Azienda Sanitaria Universitaria Friuli Centrale ASU FC, 33100 Udine, Italy; serena.dagostini@asufc.sanita.fvg.it

**Keywords:** neuropsychology, neurosurgical patients, plasticity, high-grade glioma

## Abstract

**Simple Summary:**

Studies on pre-surgery effects of high-grade glioma on cognition are few, and investigations mainly used general test batteries without specifically addressing selective neuropsychological functions. We studied the pre-surgery neuropsychological status of 85 patients with high-grade glioma, by administering several cognitive tasks to assess language, memory, executive functions, and praxis. We analyzed their lesion volumes to test anatomo-functional correlations. We found that high-grade glioma involving different sub-areas of the left temporal lobe selectively impacts cognitive functions, especially within the language domain. There was one small overlapping lesion area that was shared by all the tasks we examined, localized in the superior temporal cortex.

**Abstract:**

(1) Background: The literature on the effects of high-grade glioma (HGG) growth on cognition is still scarce. (2) Method: A consecutive series of 85 patients with HGG involving the left hemisphere underwent an extended neuropsychological evaluation prior to surgery. Voxel-based lesion-symptom mapping (VLSM) was used to identify regions related to cognitive performance. (3) Results: The patients’ mean level of pre-surgery accuracy was overall high. They showed the greatest difficulties in language with tasks such as naming (42.1% of patients impaired on nouns and 61.4% on verbs), reading (36.3% on words and 32.7% on pseudo-words), auditory lexical decisions (43.9%) and writing (41.3%) being most frequently impaired. VLSM analysis revealed anatomically separated areas along the temporal cortex and the white matter related to impairments on the different tasks, with voxels commonly shared by all tasks restricted to a small region in the ventral superior and middle temporal gyrus. (4) Conclusions: High-grade glioma affects cognition; nonetheless, lesions do not cause diffuse deficits but selectively impact the different language sub-domains along the ventral stream and the dorsal stream for language processing.

## 1. Introduction

High-grade gliomas (HGGs) are astrocytic- or oligodendrocytic-derived (WHO grade III and IV) tumors [1] with a highly malignant pattern and poor prognosis. Among all primary tumors of the central nervous system (CNS), glioblastoma multiforme is considered one of the most common and most aggressive tumor subtypes in the adult population. It has a global incidence of about 3–4 cases per 100,000 people per year [2], is more frequent in men than in women and occurs mainly in the 55–75 age group, with no particular territorial distribution, yet with a greater incidence in the Caucasian population than in African or Asian populations.

The literature on cognition in HGG is limited [3]. Motor and/or sensory alterations can be generated [4]. Cognitive deficits can have consequences upon the daily life of patients [5,6]. Patients’ complaints of symptoms occur in the weeks or months before they are admitted to a neurological or neurosurgical ward for their first examinations. Headaches, personality changes, memory disorders, motor weakness and language difficulties are among the most frequently occurring symptoms [4]. One third of patients report seizures as a major first symptom (Wychowski 2013). In general, most studies examining cognitive functioning in patients with HGG are retrospective and use neuropsychological screening batteries such as the Mini Mental State Examination. For example, impairments in executive functions, memory and attention have been reported in a group of 139 patients with frontal and temporal brain tumor evaluated a week before surgery [7]. Cognitive deficits that can occur at the onset of the disease are attributable to processes of compression, displacement, destruction, ischemia of intracranial structures or cerebral edema [8]. An analysis of pre-surgery cognitive performance in a group of 8 subjects with HGG in frontal and temporo-occipital areas showed an impairment in at least two cognitive domains [8]. In particular, 88% of patients displayed an impairment in information processing speed, executive functions as well as short-term memory. In another study [9], 196 patients with brain tumors were assessed pre- and post-craniotomy, and at follow-up. Pre-surgery assessment (1–2 days before craniotomy) showed cognitive deficits. Impairments were found in working memory, attention and in subcomponents of executive functions. Patients with HGG performed worse than patients with more favorable diagnoses (low-grade glioma or metastases). However, the cognitive deficit does not necessarily appear to be related to the location of the tumor as it seems determined by global damage [9].

The goal of our study was to evaluate cognition in a group of patients with left hemisphere HGG. In particular, we focused on framing the pre-operative cognitive status concomitant with HGG and testing whether (and to what extent) functional impairment correlates with lesion location.

## 2. Materials and Methods

### 2.1. Study Population

We retrospectively reviewed a consecutive series of 85 adult patients (age ≥ 18 years) meeting the following inclusion criteria: admission to a neurosurgical ward for a left-hemisphere HGG in the time period between 2007 and 2019 and confirmed diagnosis based on the 2016 WHO classification criteria [1]; having performed a neuropsychological evaluation; being native Italian speakers; having normal or corrected-to-normal vision; no history of psychiatric disease or drug abuse. We excluded patients with developmental language problems or learning disabilities or with a family history for such disabilities. Patients had a mean age of 57.68 years (SD = 10.72 years) and a mean education of 11.36 years (SD = 4.18 years, see Table 1). Patients were right-handed [10]. Conventional T1-weighted MR imaging was acquired before surgery and revealed high-grade neoplastic lesions (43.74, SD = 38.57 mean mm3 in volume). The lesion overlap of all 85 patients covered the left frontal, parietal and temporal areas. We classified patients according to their T1 and T2 MRI images in order to establish whether or not the lesion caused edema. Histological data was retrospectively analyzed according to the 2016 WHO classification [1] and confirmed the diagnosis of HGG.

The present study was approved by the local Ethics Committee (protocol N. 0036566/P/GEN/EGAS, ID study 2538) and performed in accordance with the 2013 Fortaleza version of the Helsinki Declaration and subsequent amendments. Written informed consent for surgery was obtained. Considering that the study was retrospective, written consent to participate in the study was not applicable.

### 2.2. Neuropsychological Assessment

All patients received a neuropsychological assessment by a neuropsychologist a few days before surgery, on the same day as the MRI. Several cognitive domains were assessed. Fluid Intelligence was assessed by Raven Matrices [11]. Executive functions were tested by the backward version of the Digit span test [12] as a measure of working memory. Praxis was tested by two imitation tasks for arm/hand (ideomotor apraxia [13]) and oro-facial (Oral Praxis [14]) movements. Short-term memory was assessed by a digit span task [12]. Language was tested by using the Battery for the Assessment of Aphasic Disorders [15]. In particular, we used the following tasks: word and pseudoword reading [15], word and pseudoword repetition [15], noun naming and verb naming [15], phonological discrimination [15], writing [15], objects auditory comprehension and verbs auditory comprehension [15], auditory lexical decision [15]. Auditory verbal comprehension was tested by using the Token test [16]. Verbal fluency was tested by a phonological fluency task [17] and a semantic fluency test [17]. Patients performed different neuropsychological tests, because new tests were added to our neuropsychological protocol during the 2008–2019 time period and because tests were selected based on tumor location. We opted for this approach as neuropsychological investigation in patients—who are often fatigued and with deficits—must be as functional as possible.

#### Neuropsychological Data Analyses

To define whether patients scored within or below the normal range, their performance on Raven Matrices, Digit span, working memory and phonological fluency was corrected for age, education and sex according to the published norms. We then calculated the corresponding equivalent scores (i.e., an equivalent score of 0 means a pathological performance). For all the other tests for which normed conversion of raw scores into equivalent scores is not available, scores were compared with the published cut-off value.

### 2.3. MRI Structural Data

Tumor topographic and volumetric descriptions were obtained by retrospectively analyzing structural imaging data routinely acquired during pre-surgery investigations. A 3-T Philips Achieva whole-body scanner was used to acquire structural data using a SENSE-Head-8 channel head coil. High-resolution T2-weighted and post-gadolinium contrast T1-weighted anatomical MR images were acquired by using a T1-weighted 3D magnetization-prepared rapid acquisition gradient fast field echo (T1W_3D_TFE SENSE) pulse sequence (TR = 8.2 s, TE = 3.7 s, FOV = 24 cm, 190 sagittal slices of 1 mm thickness, flip angle = 8°, voxel size: 1 × 1 × 1 mm^3^) and a T2-weighted 3D magnetization-prepared rapid acquisition gradient fast field echo (T2W_3D_TFE SENSE) pulse sequence (TR = 2500 ms, TE = 35 ms, FOV = 24 cm, 190 sagittal slices of 1 mm thickness, flip angle = 90°, voxel size: 1 × 1 × 1 mm^3^).

Volumes of interest (VOIs) of the patients’ lesions were drawn on their T1 MRI scans using MRIcron software, v. 1.0.20190902 (https://www.nitrc.org/projects/mricron). We then normalized the ROIs to the Montreal Neurological Institute (MNI) space using the “Clinical Toolbox” (https://www.nitrc.org/projects/clinicaltbx/) for SPM12 (https://www.fil.ion.ucl.ac.uk/spm/), by applying the normalization procedure of the toolbox. Normalization output was visually inspected to exclude that the normalization procedure was inaccurate due to possible mass effects and tissue displacements that HGG can create. 

#### 2.3.1. Voxel-Based Lesion-Symptom Mapping

VOI and behavioral data were analyzed in a voxel-based lesion-symptom mapping (VLSM) procedure. NPM (non-parametric mapping, https://www.nitrc.org/projects/mricron) software was used. We set the non-parametric Brunner Munzel (BM) test and 1000 permutations, ignoring voxels damaged in less than 10% patients. Each patient’s score on the cognitive task was used as continuous behavioral variable. The critical z-value considered as our BM map was *p* < *0*.05. Any value in the power map (see Supplementary Figure S1 showing areas taken into consideration in the VLSM analyses) and the BM map exceeding this critical z-value was considered significant.

#### 2.3.2. Further Exploratory Investigation: Subtraction Analysis

Following subtraction of lesion masks [18], we also used a subtractive approach in a limited number of patients, i.e., the subgroup of patients performing selected tasks: an overlay image was generated for tests performed by at least 40% of the sample obtaining pathological scores or by at least 20% of the sample. The subtraction approach, which had been previously adopted [19], allows for controlling for differences in sample sizes by using proportional values. For each task, we used the MRIcron procedure (https://www.nitrc.org/projects/mricron) and created lesion maps by overlapping the lesion masks (VOI) of patients with impaired performance and an overlay image of the VOIs of patients with spared performance (see Supplementary Figure S2 showing areas considered for subtraction analyses). We then used MRIcron to subtract the lesion overlay of spared patients from that of impaired patients. The output is a percentage overlay plot showing the results of subtractions for the task-impaired patients on a color scale. This % procedure, which had been previously adopted [18], allows to control for differences in sample sizes by using proportional values.

## 3. Results

We report on the patients’ clinical symptoms, their neuropsychological status, and the neural correlates of their altered neuropsychological performance.

### 3.1. Behavioral Data

#### 3.1.1. Pre-surgical Clinical Symptoms

- We analyzed the patients’ self-reported clinical signs and symptoms gleaned from case notes and from the neuropsychological interview. Patients reported clinical signs and symptoms (see Table 2). Some patients reported a combination of cognitive and motor/proprioceptive symptoms. The most frequently reported symptoms were language-related, in particular anomia and phonological paraphasia, reading and writing difficulties.

#### 3.1.2. Neuropsychological Data

The percentage of patients found to be below the cut-off for each test is shown in Table 3. All patients performed well on the Raven Matrices test, suggesting that their general intelligence was spared.

- Considering tasks performed by 80–100% of our sample (see Table 3), naming as cognitive domain showing was most impaired. Considering tasks performed by 60–80% of our sample, reading showed to be most impaired. Considering tasks performed by 40–60% of our sample, lexical decision and writing were most impaired.
- 13/85 (15%) patients had no cognitive impairments in any domain considered.
- The patients’ mean level of accuracy ranged from 80.68% to 98.21% (see Table 3), even for the cognitive domain on which the greatest percentage of patients scored below the normal range.

### 3.2. Structural Data

#### 3.2.1. Voxel-Based Lesion-Symptom Mapping (VLSM)

**Figure 1:**
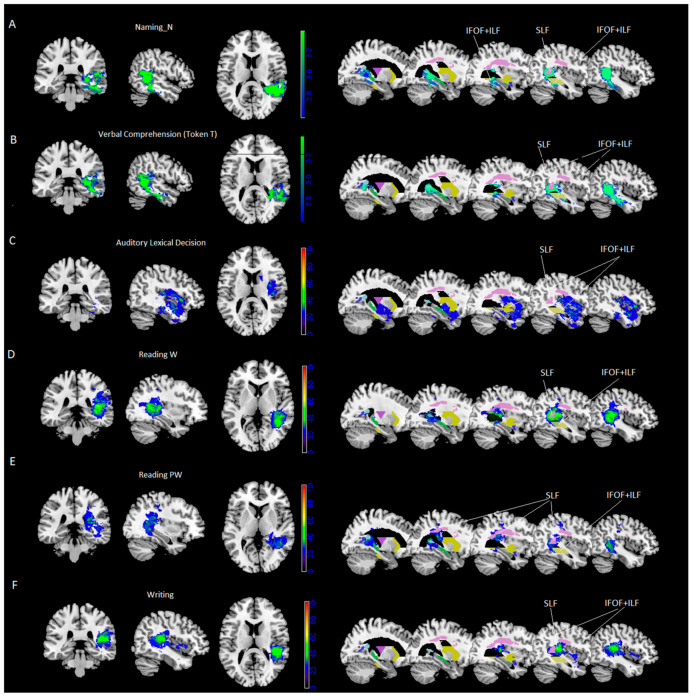
VLSM templates showing lesions significantly affecting performance on (**A**) noun naming and (**B**) token test. (*p* < 0.01, thresholds based on permutation method). Subtraction of lesion masks (**C**–**F**) of patients scoring pathologically from patients with normal performance (minimum 20% overlap) on the different tasks. MR images are displayed following radiological convention (left is right and vice versa). On the right side of the panel, results overlap with the JUH template of white matter pathways on sagittal slices. SLF = superior longitudinal fasciculus, IFOF = inferior fronto-occipital fasciculus; ILF = inferior longitudinal fasciculus belonging to sagittal stratum. N = nouns; W = words; PW = pseudowords; Token T = Token test.

- Results of VLSM analyses were significant for noun naming and verbal comprehension (Token Test) only. VLSM performed for noun naming revealed 24% of the voxels damaged in the middle temporal cortex and, to a lesser extent (between 15% and 10%), in the inferior and superior temporal gyrus, the hippocampus/parahippocampal area and the fusiform gyrus (Figure 1A). In addition, at subcortical level we found portions of the posterior thalamic radiation and the sagittal stratum [including the inferior fronto-occipital fasciculus (IFOF) and the inferior longitudinal fasciculus (ILF)], with 40% of overlapping lesions, and to a lesser extent (% voxels damaged between 29% and 14%), the retrolenticular part of the internal capsule, the superior longitudinal fasciculus (SLF, with 50% of overlapping lesions) and pars of the tapetum (see Table 4 and Figure 1A).
- VLSM analyses performed for the Token Test revealed 26% of the voxels damaged in the middle temporal cortex and, to a lesser extent (between 15% and 10% of the voxel damaged), in the inferior and superior temporal gyrus, and the hippocampus (Figure 1B). At subcortical level we found parts of the sagittal stratum (including IFOF and ILF, with 43% of overlapping lesions), posterior corona radiate and posterior thalamic radiation and the retrolenticular part of the internal capsule, pars of the tapetum and, to a lesser extent (% voxels damaged between 26% and 5%), the superior longitudinal fasciculus (SLF, with 49% of overlapping lesions) and pars of the splenium (see Table 4 and Figure 1B).
- All other tasks did not survive correction for multiple comparisons. Therefore, further exploratory investigations were performed by using a subtraction approach.

#### 3.2.2. Further Exploratory Investigation: Subtraction Analysis Performed on the Whole Sample

**Figure 2:**
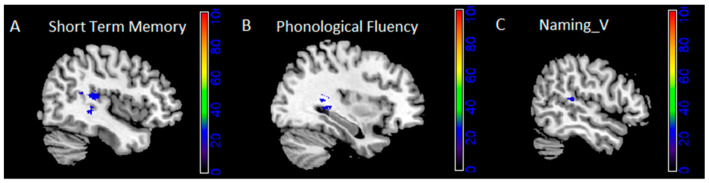
Subtraction of lesion masks of patients scoring pathologically from patients with normal performance (minimum 20% overlap) on the different tasks: short-term memory (**A**), phonological fluency (**B**) and verb naming (**C**) MR images are displayed following radiological convention (left is right and vice versa). V = verbs.

- Only tasks for which the maximum lesion overlap in the output image (lesion overlay of pathological patients—lesion overlay of spared patients) was at least >20% are reported (for a complete list of coordinates, see Supplementary Table S1). Local maxima (See Figure 2 and Table 5) were localized as follows:
- for verb naming: in the superior and middle temporal gyrus (25% and 24%);
- for phonological fluency: in the hippocampus (22%) and, at subcortical level, in the retrolenticular part of the internal capsule (26%);
- for short-term memory: in the superior temporal gyrus (30%), and, at subcortical level, in the retrolenticular part of the internal capsule (overlap: 26%) and sagittal stratum (including IFOF and ILF, 26%).

#### 3.2.3. Further Exploratory Investigation: Subtraction Analysis Performed on Part of the Whole Sample

Further exploratory subtraction analyses were performed on a part of the whole sample. Local maxima (See Figure 1C–F and Table 5) were localized as follows:

- for word reading, performed by 55/85 patients: in the superior temporal gyrus (57%) and, at subcortical level, in the superior longitudinal fasciculus (57%), the posterior thalamic radiation and the retrolenticular part of the internal capsule (47% and 44%) and the sagittal stratum (including IFOF and ILF, 36%);
- for pseudo-word reading, performed by 55/85 patients: in the calcarine cortex and the middle and inferior temporal gyrus (37%) and, at subcortical level, in the posterior thalamic radiation (39%), in the SLF (37%) and the retrolenticular part of the internal capsule and the tapetum (37%);
- for auditory lexical decision, performed by 41/85 patients: in the superior temporal gyrus (40%) and, at subcortical level, in the sagittal stratum (including IFOF and ILF, 36%);
- for writing, performed by 46/85 patients: in the superior temporal gyrus (58%) and, at subcortical level, in the SLF (45%).

#### 3.2.4. Further Investigations: Intersection Area

**Figure 3:**
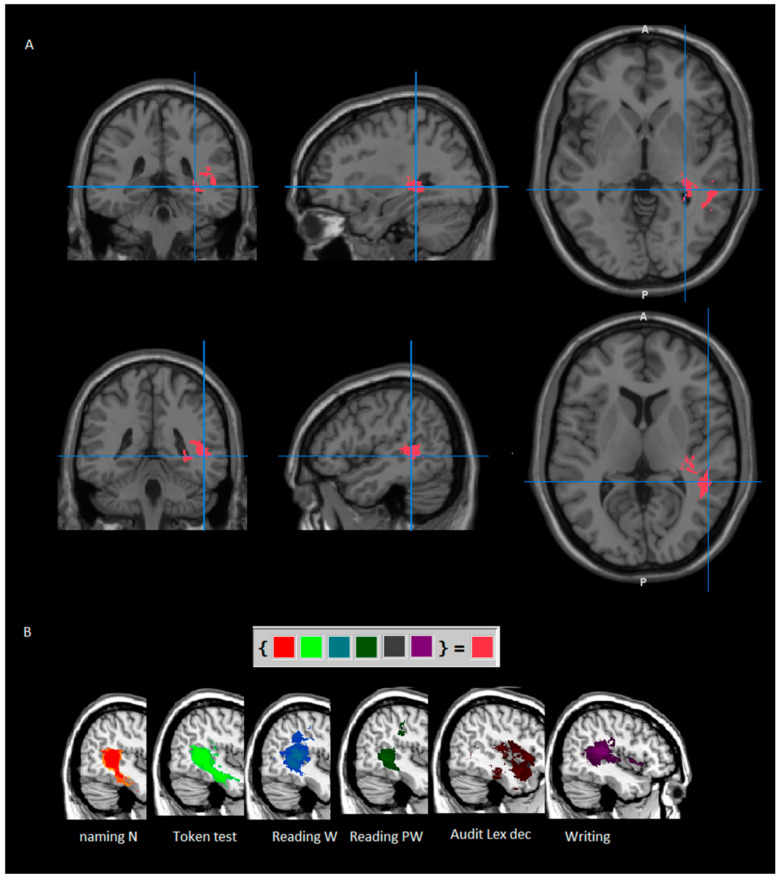
Intersection (**A**) of lesion masks of the different tasks (**B**). MR images are displayed following radiological convention (left is right and vice versa). N = nouns; W = words; PW = pseudowords; Audit lex dec = auditory lexical decision.

The Create Overlay Logicals of Mango software, version 4.1 (http://ric.uthscsa.edu/mango/mango.html) was used to trace the intersection area between the maps obtained in the previous analyses reported above, namely, naming (nouns and verbs), reading (words and pseudowords), auditory lexical decisions, and writing. The common small area was located at the intersection of part of the middle and superior temporal gyrus and, at subcortical level, it marginally involved the retrolenticular part of the corona radiate, the superior longitudinal fasciculus and the sagittal stratum (see Figure 3).

## 4. Discussion

The literature on cognition in HGG is limited [3] as most of the studies examining cognition in patients with brain tumors involved subjects with low-grade glioma [6]. We studied a consecutive series of 85 patients with HGG involving the left hemisphere. Importantly, in our retrospective analysis we investigated the cognitive framework prior to resection and radio- or chemotherapy treatments.

The study of neuropsychological status in HGG is worth conducting for several reasons. The growing pattern of HGG as compared to low-grade glioma is faster, leaving few possibilities for pre-surgery plastic reorganization. It usually causes a deficit at the very beginning, enabling understanding of anatomo-functional correlations pre-surgically. Lastly, brain tissue in HGG is already structurally impacted preoperatively, meaning that the resection is more likely to damage functional areas causing a deterioration in functional abilities. It is known that gliomas can infiltrate, destroy or dislocate the function in another location. In the latter case, there is no relation between glioma location and functional impairment. VLSM allows to assess the relation between location and boundaries of the glioma and functional impairment. From a clinical perspective, this stratification of tasks based on anatomo-functional correlations in HGG patients can be informative for surgeons (by indicating which part of the brain should be spared in order to better preserve cognitive abilities).

### 4.1. Neuropsychological Results

Cognitive tasks showing the greatest impairment were naming (nouns and verbs), reading (words and pseudowords), auditory lexical decisions and writing. The other tasks, namely, verbal comprehension, phonological fluency, short-term memory and working memory and imitation of gestures, repetition, auditory verb comprehension, auditory object comprehension, phonological discrimination were less impaired. This can suggest which tasks are worth to be tested during awake surgery if such approach is planned, in order to preserve the cognitive status post-operatively as much as possible.

An analysis of pre-surgery cognitive performance in a group of 8 subjects with HGG in frontal and temporo-occipital areas showed an impairment in at least two cognitive domains, namely information processing speed and short-term memory [8]. In our sample, the mean number of tasks in which patients had an impairment was 4.5 (SD = 4). In another study [9], 196 patients with brain tumors were assessed pre-craniotomy (1–2 days before craniotomy) and showed impairments in working memory (61.4%), attention and subcomponents of executive functions. In general, most of the studies examining cognitive functioning in patients with HGG are retrospective and use general neuropsychological screening batteries such as the Mini Mental State Examination or executive functions, memory and attention [7]. By contrast, we investigated cognitive functioning with function-specific neuropsychological tests. This enabled us to detect deficits beyond the attention and/or executive functioning deficit generally detected as the most impaired component in HGG patients. Interestingly, the cognitive deficit did not necessarily appear to be related to the location of the tumor as it seems determined by global damage [9]. By contrast, in our study, the VLSM and subtraction analyses (see below) revealed that deficits were related to the location of the tumor. No post-surgery or follow-up data are presented in our study. This could be of particular interest, as follow-up data can allow to test whether neuropsychological impairments have a tendency to improve post-surgery or whether patients show a progressive decline, perhaps depending on disease progression. This issue could be the focus of future studies.

### 4.2. Neuroanatomical Results

Among cognitive tests, object naming and verbal comprehension emerged as the most sensitive to VLSM analyses (Token Test).

Impaired object naming correlated with voxels in the middle temporal cortex and, to a lesser extent, in the inferior and superior temporal gyrus, the hippocampus/parahippocampal area and the fusiform gyrus. To discuss findings at subcortical level, we used the framework of the dorsal and the ventral stream for language processing, which is (e.g., [22,23,24,25]) held to be involved in phonology and semantic aspects. The model holds that the dorsal stream, via the arcuate fasciculus, arises from the posterior superior temporal gyrus and projects to the inferior parietal lobe and to the inferior frontal gyrus and premotor cortex; the ventral stream, via the inferior fronto-occipital fasciculus, connects the superior and middle temporal gyrus to the inferior parietal lobe and occipital lobe, and to the inferior frontal gyrus (e.g., [22,23,24,25]. This model has been previously applied to the study of patients with neurosurgical lesions in the left hemisphere (e.g., [26,27,28,29,30]).

At subcortical level, we found that naming correlated with voxels in parts of the posterior thalamic radiation and the sagittal stratum (including IFOF and ILF) and, to a lesser extent, the retrolenticular part of the internal capsule, the superior longitudinal fasciculus and pars of the tapetum. Thus, both the ventral streams driving lexical-semantic processing and the dorsal streams driving phonological processing were involved as the task is a naming aloud task. This is in line with a previous VLSM study performed by our group in which we found that noun naming impairments were associated with basal temporo-occipital lesions [31]. In addition, in the [31] naming task, impairments were related to damage to parts of the sagittal stratum (including the inferior fronto-occipital fasciculus and the inferior longitudinal fasciculus), the splenium of the corpus callosum, the posterior thalamic radiations (plus optic radiations), the retrorolandic part of the internal capsule, the tapetum and the fornix. Other neuroimaging experiments, e.g., [32,33,34,35] and two meta-analyses of functional imaging studies focusing on lexical-semantic processing [32,33,36,37], showed a role of the left posterior basal occipital and temporal cortex in naming nouns.

The Token Test is used to test subtle auditory comprehension deficits. Patients are asked to respond (by touching/moving colored tokens) to verbal commands of increasing length. In a study on stroke patients [38], the authors found that comprehension (they used six comprehension scores among which the Token Test) was correlated to 51% of the fibers of the ventral stream and to 22% of the fibers of the dorsal stream. The VLSM analyses performed for the Token Test revealed a set of voxels in the posterior part of the left (middle and inferior) temporal gyri as well as the fusiform gyrus and the supramarginal gyri. In a VLSM study, Pisoni et al. [39] found that impairments on the Token Test significantly correlated with damaged voxel in the posterior part of the left superior and middle temporal gyri as well as with the angular and supramarginal gyri. At subcortical level, both the SLF and the sagittal stratum (IFOF+ILF) correlated with the task, thus involving both the dorsal and ventral stream.

To conclude, naming and verbal comprehension (Token Test) involved equally the ventral and the dorsal streams for language processing.

### 4.3. Further Exploratory Neuroanatomical Analysis

For the other tasks, no voxels survived the predefined threshold in VLSM. In this case, the subtraction analyses of lesion VOIs (% of pathological patients—% spared patients) showed that the % of overlap exceeded 40% or was just below it for reading (words and pseudo-words), auditory lexical decision and writing (overall score for words and pseudo-words). The other tests (verb naming, phonological fluency, short-term memory, working memory) showed a less consistent % of maximum overlap, suggesting that these tasks involve networks of areas different than those involved in our sample (mainly the temporal lobe) and will not be discussed any further.

Local maxima—in terms of maximum % overlay for auditory lexical decisions—involved the superior temporal gyrus and the sagittal stratum (IFOF+ILF). In the auditory lexical decision task, meaning access occurs through the acoustic-phonetic system. Previous fMRI studies showed that auditory lexical decisions activated a network of brain regions, including superior, middle temporal gyrus, calcarine and lingual gyrus, and left supramarginal gyrus and bilateral inferior frontal gyrus (e.g., [40]). In a positron emission tomography study, it was found that auditory lexical decision task compared to an auditory control condition activated bilaterally the superior temporal and inferior frontal gyrus [41]. Similar results were found in another positron emission tomography study reporting that the auditory lexical decision task activated the superior temporal gyrus ventro-laterally to Heschl’s gyrus.

Local maxima—in terms of maximum % overlay for word reading—involved the calcarine cortex, the inferior and middle temporal gyrus and, at subcortical level, both the SLF and the sagittal stratum (IFOF+ILF), while for pseudoword reading it involved the superior and middle temporal gyrus and, at subcortical level, the SLF to a greater extent than the sagittal stratum (IFOF+ILF). This result is consistent with the functional imaging literature reporting the left occipito-temporal, inferior [42], middle [43] temporal gyrus and the inferior frontal gyrus [44], the IFOF and the ILF [45,46] as correlates of the lexical-semantic route of reading. The left temporo-parietal junction [44,47,48] or a variety of perisylvian cortical regions [49] and the left arcuate fasciculus [45,46] have been found to be associated with the sublexical route. Italian is a shallow orthography language. The sublexical phonological route is sufficient for reading the great majority of words; exceptions, concerning for example word stress, must be processed along the lexical route.

Local maxima—in terms of maximum % overlay for writing—involved the superior temporal gyrus and, at subcortical level, the SLF and the sagittal stratum (IFOF+ILF, although to a lesser degree). Roux et al. [50] used direct electrical stimulation mapping during awake surgery, while patients performed a writing task. They found that the positive sites (i.e., sites at which stimulation caused interference with writing) were mostly located along the superior temporal gyrus (Brodmann’s areas 22 and 42). Other authors found that the SLF was related to dysgraphia in a neurosurgical patient [51].

To conclude, at subcortical level, we found that lexical decision involved the ventral stream (in terms of % of overlapping lesions), while reading words involved equally the ventral and the dorsal stream. Lastly, reading pseudowords and writing involved the dorsal stream. Other white matter tracts showed a high maximum % of lesion overlap: the posterior thalamic radiation and the retrolenticular part of the internal capsule. These results are in line with studies showing functional roles for these white matter tracts for language capacities (e.g., [52,53,54]).

One potential limitation of the study is the different sample size between naming nouns, naming verbs, Token test, phonological fluency and short-term memory, which were administered to the whole sample, and reading words and pseudowords, writing, and lexical decisions which were administered to part of the sample. For the latter, we interpreted our results as being exploratory. Nonetheless, the subtraction approach, which has been previously adopted by Karnath, Berger, Küker, and Rorden (2004), allows controlling for differences in sample sizes by using proportional values.

## 5. Conclusions

HGG lesions along the left (superior) temporal cortex selectively affect language abilities. Task-related lesion maps (both the VLSM and the less robust subtraction analyses results) share voxels in a small region in the ventral superior and middle temporal gyrus. At subcortical level, we found that lexical decisions involved the ventral stream, naming verbal comprehension and reading words involved the ventral and the dorsal stream, reading pseudowords and writing involved the dorsal stream for language processing.

## Figures and Tables

**Table 1:** Demographic, tumor clinical and histological data of the study group.

Parameter	Value
No. of patients	*n* = 85
Age (years)	Mean = 57.68; sd = 10.72; range 26–74 years
School (years)	Mean = 11.36; sd = 4.18; range 5–18 years
Handedness	Right = 83 patients; left = 2 patients
Sex	53 males; 33 females
Tumor volume	Mean = 43.74, sd = 38.57; range 1.43–260.15
WHO grade	III = 8 patients; IV = 77 patients
Edema	Present = 44 patients; absent = 41 patients

**Table 2:** Pre-surgery signs and symptoms.

Signs and Symptoms	No. of Patients	Percentages Positive for Each Symptom	95% Confidence Interval Lower	95% Confidence Interval Upper
Language disorders (anomia, paraphasia, writing and reading)	36	42.35	32	53
Tingling (face and/or limbs)	9	10.59	4	17
Seizures	13	15.29	7	23
Emotional lability	1	1.18	−1	4
Memory difficulty	3	3.53	0	8
Attention deficit	2	2.35	−1	6
Clonia (face and/or limbs)	7	8.24	2	14
Vomiting	3	3.53	0	8
Ear disorders	1	1.18	−1	4
Dizziness and/or head turns	2	2.35	−1	6
Visual disturbances (hempianopsia)	4	4.71	0	9
Loss of balance	1	1.18	−1	4
Strength loss	5	5.88	0	11
Headaches	6	7.06	2	13
Paresis	2	2.35	−1	6
Agitation	1	1.18	−1	4
Praxis deficit	3	3.53	0	8
Confusion	9	10.59	4	1.7
Cacosmia or perception of perfumes	2	2.35	−1	6

**Table 3:** Neuropsychological performance (accuracy) of patients with left-hemisphere high-grade glioma (HGG).

Test	No. of Patients Performing the Task	% Pathological	Accuracy Mean % or Mean Performance	SD	Min	Max	95% Confidence Interval Lower	95% Confidence Interval Upper
Verb naming	85	61.45	82.27	17.97	17.86	100.00	78.34	86.19
Object naming	85	42.17	85.50	20.05	3.33	100.00	81.12	89.88
Ideomotor apraxia	85	10.98	92.08	15.97	8.33	100.00	88.54	95.6
Oral Praxis	85	2.44	97.20	8.43	40.00	100.00	95.34	99.04
Token Test	85	23.75	80.68	18.97	9.72	100.00	76.45	84.89
Digit span forward	79	36.71	4.81	1.05	2.77	7.23	4.56	5.03
Raven Matrices	85	0.00	83.76	10.26	62.5	100.00	81.44	86.07
Phonological Fluency	76	36.84	24.1	13	1	52	20.75	26.79
Working Memory	75	37.33	3.07	0.93	1.5	5.64	2.85	3.28
Pseudoword repetition	57	21.05	94.35	16.22	0.10	100.00	90.04	98.65
Words repetition	57	14.04	96.90	13.87	0.10	100.00	93.22	100.58
Word reading	55	36.36	93.17	16.35	0.10	100.00	88.75	97.59
Pseudoword reading	55	34.55	87.11	23.97	0.10	100.00	80.63	93.59
Word and pseudoword writing	46	41.30	89.33	14.28	24.00	100.00	85.08	93.56
Auditory comprehension: verb	43	9.30	97.20	5.61	85.00	100.00	96.92	99.35
Auditory comprehension: object	42	14.29	98.14	3.94	72.50	100.00	95.45	98.95
Phonological Discrimination	42	2.38	97.74	13.36	13.33	100.00	93.57	101.9
Auditory lexical decision	41	48.78	91.33	19.52	1.57	100.00	84.99	97.65

**Table 4:** Results of the VLSM analysis on naming and verbal comprehension (Token test).

Area ^1^	No. of Voxels	% of tot	Max Z-Score	x	y	z
**Naming**						
Superior Temporal Gyrus	2716	10.75	7.15	43	−38	12
Middle Temporal Gyrus	8509	23.97	3.89	49	−35	−13
Inferior Temporal Gyrus	3671	12.89	3.89	53	−13	−32
Fusiform gyrus	2684	13.26	3.89	44	−31	−17
Hippocampus	1160	15.25	3.89	31	−36	−4
Parahippocampal area	996	11.03	3.89	33	−40	−7
Precuneus	274	1.05	3.71	27	−49	5
Calcarine cortex	454	3.05	3.71	26	−48	6
Supramarginal gyrus	126	0.79	3.61	57	−25	19
Lingual gyrus	121	0.65	3.61	27	−45	−3
Middle frontal gyrus	83	0.2	2.84	33	12	37
Posterior thalamic radiation	1912	48	3.89	34	−47	−3
Sagittal stratum (IFOF+ILF)	1041	46	3.89	41	−34	−14
Superior longitudinal fasciculus	950	14	6.12	40	−41	14
Retrolenticular part of the internal capsule	725	29	3.89	35	−38	−3
Fornix	257	22	3.89	29	−29	−4
Tapetum	257	42	3.89	30	−44	8
**Token Test**						
Superior temporal gyrus	2577	10.21	3.89	43	−41	5
Middle temporal gyrus	9295	26.19	3.89	49	−35	−13
Inferior temporal gyrus	4503	15.81	4.83	45	−21	−20
Fusiform gyrus	1606	7.93	5.11	44	−24	−16
Hippocampus	1038	13.64	4.31	37	−31	−6
Parahippocampal area	306	3.38	3.61	35	−41	−4
Calcarine cortex	829	5.56	3.89	27	−50	5
Supramarginal gyrus	32	0.21	2.86	45	−34	24
Lingual gyrus	86	0.46	3.71	23	−51	4
Precuneus	221	0.84	3.89	27	−47	5
Superior temporal pole	134	1.25	3.01	61	4	−8
Heschl’s gyrus	43	2.22	3.41	39	−26	9
Rolandic operculum	21	0.19	2.91	45	−29	20
Posterior thalamic radiation	2070	52	3.89	37	−42	−3
Sagittal stratum (IFOF+ILF)	1416	63	4.31	37	−31	−6
Retrolenticular part of the internal capsule	1074	43	5.15	38	−31	−1
Superior longitudinal fasciculus	840	12	3.89	41	−48	2
Tapetum	285	47	3.89	28	−48	7
Fornix	59	5	3.35	29	−32	−1
Splenium of the corpus callosum	34	26	3.13	21	−54	8
Posterior corona radiata	18	48	3.19	29	−40	19

^1^ Anatomical localization of the maximum lesion overlays was based on the automated anatomical atlas (AAL) [20] as reference anatomical atlas and, at subcortical level, the Johns Hopkins University (JHU) Diffusion Tensor Imaging (DTI)-based white-matter atlases [21]. VLSM: voxel-based lesion-symptom mapping; IFOF: inferior fronto-occipital fasciculus; ILF: inferior longitudinal fasciculus.

**Table 5:** Subtraction analysis was performed for each cognitive test. Local maxima are reported (Max).

Area	% Overlap	x	y	z
**Reading pseudowords**				
Calcarine gyrus	37	27	−52	11
Middle temporal gyrus	37	45	−48	−3
Inferior temporal gyrus	37	47	−45	−4
Posterior thalamic radiation	39	34	−46	10
Retrolenticular part of the internal capsule	37	30	−37	13
Posterior corona radiata	37	27	−42	19
Superior longitudinal fasciculus	37	37	−52	14
Tapetum	37	27	−52	11
**Reading words**				
Calcarine gyrus	37	27	−52	11
Middle temporal gyrus	37	45	−48	−3
Inferior temporal gyrus	37	47	−45	−4
Posterior thalamic radiation	39	34	−46	10
Retrolenticular part of the internal capsule	37	30	−37	13
Posterior corona radiata	37	27	−42	19
**Auditory lexical decision**				
Calcarine gyrus	37	27	−52	11
Middle temporal gyrus	37	45	−48	−3
Inferior temporal gyrus	37	47	−45	−4
Posterior thalamic radiation	39	34	−46	10
**Writing**				
Calcarine gyrus	37	27	−52	11
Middle temporal gyrus	37	45	−48	−3
**Short-term memory**				
Calcarine gyrus	37	27	−52	11
Middle temporal gyrus	37	45	−48	−3
Inferior temporal gyrus	37	47	−45	−4
**Verb naming**				
Calcarine gyrus	37	27	−52	11
Middle temporal gyrus	37	45	−48	−3
**Phonological fluency**				
Calcarine gyrus	37	27	−52	11
Middle temporal gyrus	37	45	−48	−3

## Data Availability

The data that support the findings of this study are available on request from the corresponding author. The data are not publicly available due to privacy or ethical restrictions.

## Supplementary Materials

The following is available online at https://www.mdpi.com/2072-6694/13/6/1467/s1, Figure S1: Power map, Figure S2: Overlaps of lesion masks of patients with spared (on the left side of the panel) and impaired (on the right side of the panel) performance, Table S1: Subtraction analysis performed for each cognitive test.
