# Supplementary material for: Pre-Surgery Cognitive Performance and Voxel-Based Lesion-Symptom Mapping in Patients with Left High-Grade Glioma
Source: Cancers (Basel). 2021 Mar 23;13(6):1467. doi: 10.3390/cancers13061467 (PMC8004913; doi:10.3390/cancers13061467)
Supplement: Supplementary file 1 [file cancers-13-01467-s001.pdf]

cancers

# Supplementary Material: Pre-Surgery Cognitive Performance and Voxel-Based Lesion-Symptom MAPPING in Patients with Left High-Grade Glioma

Ilaria Guarracino, Tamara Ius, Cinzia Baiano, Serena D'Agostini, Miran Skrap and Barbara Tomasino

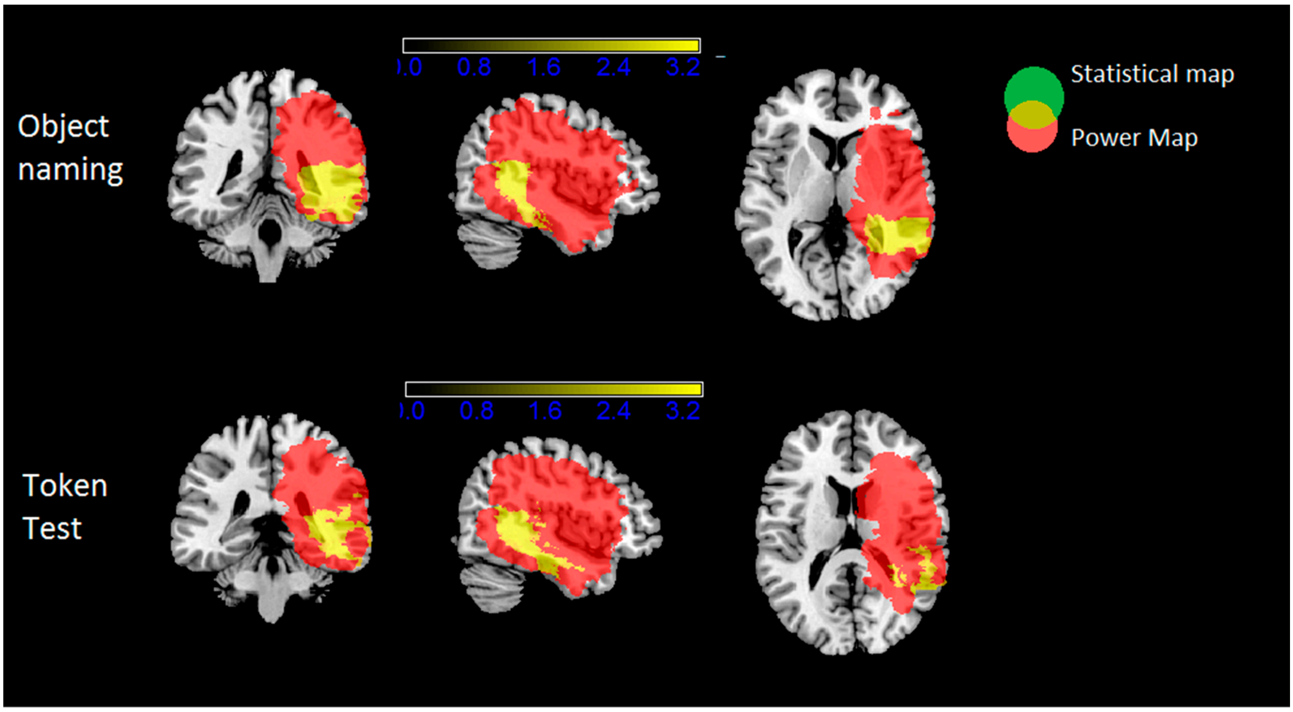


**Figure S1.** Power map. Maps of statistical power for each of the two tasks. Red areas have sufficient statistical power to detect a significant effect at $p < 0.05$ (FDR), as calculated by the Non-Parametric Mapping (see Methods for details). Yellow areas show the overlap of statistical VLSM results at P < 0.05 (FDR) with the power map.

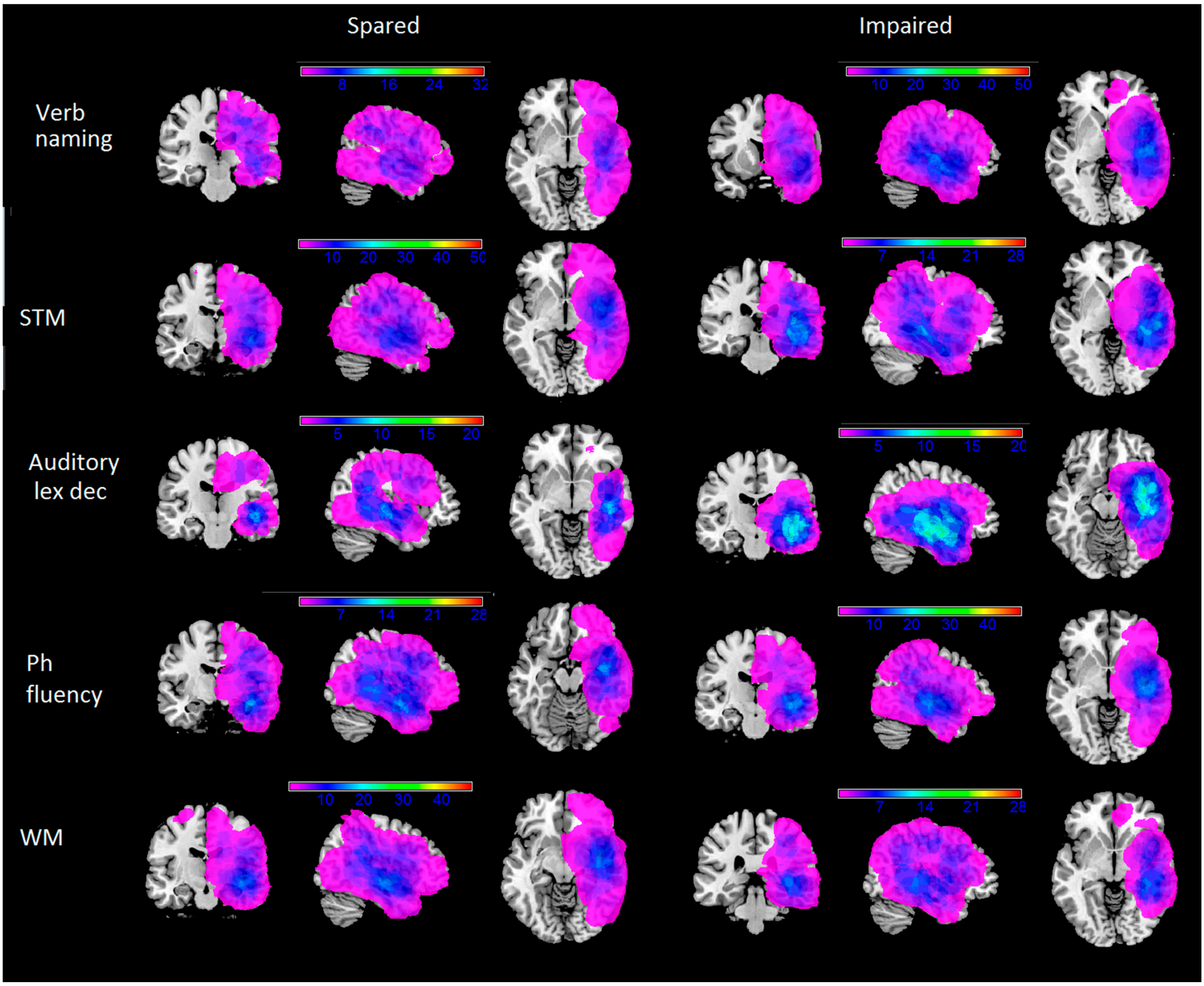


**Figure S2.** Overlaps of lesion masks of patients with spared (on the left side of the panel) and impaired (on the right side of the panel) performance. MR images are given in radiological convention (right is left and vice versa). The output is an overlay plot showing on a color scale the percentage of overlapping lesions.

**Table S1.** Subtraction analysis was performed for each cognitive test.

| **Reading P** | | | | |
|---|---|---|---|---|
| Area | Max | MaxX | MaxY | MaxZ |
| Calcarine gyrus | 37 | 27 | −52 | 11 |
| Middle temporal gyrus | 37 | 45 | −48 | −3 |
| Inferior temporal gyrus | 37 | 47 | −45 | −4 |
| Fusiform gyrus | 34 | 40 | −42 | −13 |
| Superior temporal gyrus | 33 | 36 | −32 | 12 |
| Precuneus | 32 | 27 | −49 | 11 |
| Lingual gyrus | 29 | 23 | −51 | 4 |
| Hippocampus | 29 | 28 | −35 | −2 |
| Heschl's gyrus | 29 | 33 | −28 | 16 |
| Postcentral gyrus | 29 | 44 | −26 | 45 |
| ParaHippocampal gyrus | 28 | 35 | −41 | −4 |
| Thalamus | 26 | 24 | −23 | 13 |
| Supramarginal gyrus | 26 | 49 | −30 | 33 |
| Rolandic Operculum | 24 | 25 | −23 | 20 |
| Insula | 24 | 32 | −25 | 18 |
| Inferior parietal gyrus | 21 | 38 | −38 | 45 |
| Angular gyrus | 21 | 31 | −45 | 39 |
| Posterior thalamic radiation | 39 | 34 | −46 | 10 |
| Retrolenticular part of the corona radiata | 37 | 30 | −37 | 13 |
| Posterior corona radiata | 37 | 27 | −42 | 19 |
| Superior longitudinal fasciculus | 37 | 37 | −52 | 14 |
| Tapetum | 37 | 27 | −52 | 11 |
| Sagittal stratum (IFOF+ILF) | 34 | 40 | −40 | −6 |
| Posterior limb of the internal capsule | 29 | 26 | −23 | 13 |
| Splenium | 26 | 18 | −48 | 8 |
| External capsule | 26 | 28 | −22 | 12 |
| Fornix | 26 | 23 | −32 | 7 |
| Superior corona radiata | 24 | 24 | −23 | 19 |
| Reading W | | | | |
| Superior temporal gyrus | 57 | 43 | −38 | 12 |
| Middle temporal gyrus | 52 | 37 | −50 | 14 |
| Heschl's gyrus | 46 | 36 | −31 | 13 |
| Inferior temporal gyrus | 39 | 47 | −46 | −3 |
| Calcarine cortex | 37 | 30 | −51 | 12 |
| Precuneus | 37 | 30 | −49 | 11 |
| Fusiform gyyrus | 36 | 33 | −44 | −3 |
| Hippocampus | 34 | 33 | −36 | 0 |
| ParaHippocampal gyrus | 34 | 35 | −41 | −4 |
| Lingual gyrus | 34 | 30 | −46 | −5 |
| SupraMarginal gyrus | 32 | 47 | −42 | 22 |
| Rolandic operculum | 29 | 37 | −31 | 19 |
| Angular gyrus | 29 | 41 | −48 | 23 |
| Inferior parietal gyrus | 27 | 49 | −43 | 39 |
| Postcentral gyrus | 24 | 46 | −23 | 32 |
| Insula | 24 | 35 | −29 | 19 |
| Superior longitudinal fasciculus | 57 | 40 | −42 | 15 |
| Posterior thalamic radiation | 47 | 34 | −45 | 9 |
| Retrolenticular part of the corona radiata | 44 | 34 | −34 | 7 |
| Sagittal stratum (IFOF+ILF) | 36 | 37 | −41 | −5 |
| Tapetum | 32 | 28 | −52 | 11 |
| Fornix | 31 | 29 | −32 | −1 |

| | | | | |
|---|---|---|---|---|
| Auditory Lexical decision | | | | |
| Superior temporal gyrus | 40 | 59 | −9 | −7 |
| Insula | 36 | 41 | −1 | −13 |
| Hippocampus | 36 | 40 | −20 | −17 |
| Roladic_Oper | 35 | 38 | −4 | 12 |
| Amygdala | 35 | 32 | −1 | −18 |
| Fusiform | 35 | 30 | −29 | −15 |
| Caudate | 35 | 40 | −29 | −15 |
| Hrschl | 35 | 48 | −12 | 9 |
| Superior temporal pole | 35 | 45 | 3 | −18 |
| Middle temporal gyrus | 35 | 45 | 3 | −22 |
| Temporal_Pole | 35 | 47 | 4 | −17 |
| ParaHippocampal gyrus | 31 | 37 | −19 | −21 |
| Inferior temporal gyrus | 31 | 43 | −21 | −20 |
| Inferior frontal gyrus (opercular part) | 30 | 50 | 12 | 0 |
| Inferior frontal gyrus (orbital part) | 30 | 36 | 17 | −21 |
| Palludum | 30 | 29 | −12 | −4 |
| Inferior frontal gyrus (triangular part) | 25 | 46 | 21 | 0 |
| Olfactory | 25 | 28 | 12 | −19 |
| Thalamus | 25 | 22 | −19 | −1 |
| Saggital stratum (IFOF+ILF) | 36 | 40 | −21 | −16 |
| External_capsule | 35 | 33 | −12 | −1 |
| Uncinate fasciculus | 35 | 36 | 1 | −21 |
| Retrolenticular part of the corona radiata | 30 | 30 | −21 | −2 |
| Fornix | 26 | 30 | −8 | −18 |
| Posterior limb of the internal capsule | 25 | 21 | −19 | −4 |
| Writing | | | | |
| Superior temporal gyrus | 58 | 42 | −28 | 11 |
| Heschl | 54 | 36 | −31 | 13 |
| Rolandic Operculum | 42 | 44 | −31 | 19 |
| Rolandic Operculum | 42 | 44 | −31 | 19 |
| Middle temporal gyrus | 42 | 46 | −43 | 12 |
| SupraMarginal | 38 | 46 | −34 | 24 |
| Insula | 37 | 36 | −29 | 19 |
| Hippocampus | 26 | 33 | −36 | 0 |
| Caudate | 26 | 16 | 6 | 19 |
| Putamen | 24 | 35 | −3 | −1 |
| Superior temporal pole | 24 | 39 | 9 | −26 |
| ParaHippocampal gyrus | 22 | 32 | −39 | −5 |
| Fusiform | 22 | 33 | −40 | −3 |
| Inferior temporal gyrus | 22 | 47 | −46 | −3 |
| Frontal_sup | 21 | 23 | 18 | 38 |
| Frontal_Sup | 21 | 23 | 18 | 38 |
| Superior longitudinal fasciculus | 45 | 39 | −41 | 14 |
| Retrolenticular part of the corona radiata | 36 | 35 | −34 | 6 |
| Posterior thalamic radiation | 31 | 38 | −39 | −2 |
| Superior corona radiata | 26 | 22 | 1 | 22 |
| Sagittal stratum (IFOF+ILF) | 26 | 38 | −36 | −4 |
| Superior fronto occipital fasciculus | 26 | 18 | 8 | 19 |
| Short term memory | | | | |
| Superior temporal gyrus | 30 | 41 | −32 | 13 |
| Middle temporal gyrus | 28 | 41 | −32 | 13 |
| Heschl's gyrus | 26 | 38 | −26 | 12 |
| Hippocampus | 24 | 34 | −28 | −6 |

| | | | | |
|---|---|---|---|---|
| Fusiform gyrus | 24 | 38 | −26 | −17 |
| Rolandic Operculum | 22 | 43 | −30 | 19 |
| ParaHippocampal gyrus | 22 | 28 | −42 | −3 |
| Inferior temporal gyrus | 22 | 54 | −24 | −4 |
| Lingual gyrus | 21 | 29 | −43 | −6 |
| Retrolenticular part of the corona radiata | 26 | 39 | −32 | −3 |
| Sagittal stratum (IFOF+ILF) | 26 | 39 | −30 | −12 |
| Verb naming | | | | |
| Superior temporal gyrus | 25 | 47 | −39 | 11 |
| Middle temporal gyrus | 24 | 47 | −40 | 11 |
| Inferior temporal gyrus_ | 20 | 56 | −15 | −23 |
| Phonological Fluency | | | | |
| Hippocampus | 22 | 31 | −37 | 3 |
| Precuneus | 21 | 28 | −47 | 5 |
| Retrolenticular part of the corona radiata | 26 | 33 | −37 | 3 |
| Posterior thalamic radiation | 24 | 38 | −39 | −2 |
